# Supplementary material for: Comparative Analysis of the Antimicrobial Activity of Essential Oils and Their Formulated Microemulsions against Foodborne Pathogens and Spoilage Bacteria
Source: Antibiotics (Basel). 2022 Mar 25;11(4):447. doi: 10.3390/antibiotics11040447 (PMC9025571; doi:10.3390/antibiotics11040447)
Supplement: Supplementary file 1 [file antibiotics-11-00447-s001.zip › antibiotics-1650172-supplementary.pdf]

Supplementary Material

Comparative analysis of the antimicrobial activity of essential oils and their formulated microemulsions against foodborne pathogens and spoilage bacteria

Raffaella Campana[1*], Mattia Tiboni[1], Filippo Maggi[2], Loredana Cappellacci[2], Kevin Cianfaglione[3],

Mohammad Reza Morshedloo[4], Emanuela Frangipani[1], Luca Casettari[1]

[1] Department of Biomolecular Science, University of Urbino, 61029 Urbino, Italy

[2] School of Pharmacy, University of Camerino, 62032 Camerino, Italy

[3] UMR UL/AgroParisTech/INRAE 1434 Silva, Université de Lorraine, Faculté des Sciences et Techniques - BP 70239 – 54506 Vandoeuvre-lès-Nancy, France

[4] Department of Horticultural Science, Faculty of Agriculture, University of Maragheh, Maragheh, 55136-553, Iran

*Corresponding author:

Dr. Raffaella Campana, PhD,

Department of Biomolecular Sciences

Division of Pharmacology and Hygiene

Via S. Chiara 27, 61029 Urbino, Italy

Tel: +39 0722 303543

Fax: +39 0722 303541

e-mail: raffaella.campana@uniurb.it

Table S1. Chemical composition of the essential oils investigated in this study by gas chromatography.

Component	RI[a]	RI lit.[b]						(%)[c]					
			C.sa.[d]	*C.ca.*	*C.ma.*	*C.cy.*	*C.le.*	*C.ar.*	*F.a.f.*	*F.gu.*	*J.co*	*J.pf.*	*P.an.*
Bornylene	904	907										Tr[e]	
Santolina triene	906	906					Tr						
Tricyclene	919	921					0.1	0.3			0.1	2.3	
α-Thujene	921	924	0.1		0.4	0.2	0.9	0.7		5.0	3.7	0.4	
α-Pinene	926	932	10.8	0.7	6.4	0.5	16.7	41.0	0.2	10.6	32.8	2.1	
α-Fenchene	940	945					0.9	0.1					
Camphene	940	946	0.1		Tr	Tr	0.1	0.4		0.1	0.2	1.8	
Thuja-2,4(10)-diene	948	953						0.1			0.1		
Sabinene	969	969			6.0	0.5	10.4	2.7		2.1	22.1	5.4	
β-Pinene	968	974	4.0	0.5	0.3	9.8	1.2	1.1	0.6	38.0	1.3	0.2	
1-Octen-3-ol	981	974					0.2	Tr			Tr	Tr	
Myrcene	990	988	38.5		0.5	0.6	2.6	3.6	Tr	1.6	2.3	7.6	
3-Octanol	1001	991											
δ-2-Carene	1002	1001									0.1	0.2	
α-Phellandrene	1003	1002	0.5		Tr	0.2	0.1	0.1		0.4	0.6	Tr	
δ-3-Carene	1008	1008	0.2		Tr	Tr	20.5	0.9		Tr	0.1		
α-Terpinene	1014	1014	0.3		0.7	0.1	2.1	0.9		Tr	1.9	0.5	
1-*p*-menthene	1020	1021				Tr							
o-Cymene	1022	1022					Tr						
p-Cymene	1023	1020		0.8	8.7	10.2	0.9	1.7		1.0	3.0	0.2	
Limonene	1025	1024	5.4	46.7	Tr	0.4	15.4	7.4			Tr	6.9	
β-Phellandrene	1026	1025			0.2	0.4				20.9	5.0		
1,8-Cineole	1028	1026	0.1			0.2		1.1					
(*Z*)-β-Ocimene	1037	1032	0.1		1.0			Tr	0.4	0.8			
(*E*)-β-Ocimene	1047	1044	3.7					Tr	1.1	0.2	0.1	0.3	
γ-Terpinene	1055	1054	0.2		32.9	12.2	3.5	1.4		0.2	3.3	0.9	
cis-Sabinene hydrate	1067	1065			Tr	Tr	0.7	Tr			0.2	0.1	
cis-Linalool oxide	1073	1067										Tr	
Butyl angelate	1083	1085					Tr						
p-Mentha-2,4(8)-diene	1085	1085					0.1						
Terpinolene	1085	1086	17.0		0.2	Tr	3.1	0.8			1.3	0.6	
6-Camphenone	1088	1095									Tr		
p-Cymenene	1091	1089						0.1			0.1		

2-Nonanone	1097	1096				Tr					
trans-Sabinene hydrate	1098	1098		0.1	0.2				0.2	Tr	
Linalool	1101	1095	Tr	Tr	Tr	0.3				0.6	
Isopentyl isovalerate	1106	1102							0.1		
2-Methyl butyl isovalerate	1110	1103			0.1						
trans-Thujone	1116	1112				Tr			Tr	3.8	
cis-*p*-Menth-2-en-1-ol	1121	1118		Tr	0.4	0.1			0.3	0.1	
α-Campholenal	1126	1122			Tr	0.1			0.2		
allo-Ocimene	1131	1128	Tr								
cis-Limonene oxide	1134	1132			0.1						
iso-3-Thujanol	1135	1134								0.1	
trans-Pinocarveol	1135	1135		0.1		0.2			0.4		
trans-Sabinol	1135	1137			Tr					2	
trans-Limonene oxide	1138	1137			Tr	Tr					
trans-*p*-Menth-2-en-1-ol	1139	1136			0.3	0.1					
Geijerene	1139	1138									Tr
trans-Verbenol	1140	1140							0.3		
Camphor	1141	1141			0.1	1.9				0.2	
Camphene hydrate	1144	1145				0.1				0.3	
Citronellal	1156	1148			0.1						
Sabina ketone	1158	1154				Tr			0.2		
Pinocarvone	1159	1160		Tr							
Borneol	1165	1165			0.1	0.1			0.1	1.2	
(*Z*)-*sec*-Butyl propenyl disulfide	1166	1168					39.2				
p-Mentha-1,5-dien-8-ol	1167	1168			0.1	0.2			0.3		
Coahuilensol	1171	1166								0.1	
(*E*)-*sec*-*B*utyl propenyl disulfide	1171	1174					26.3				
Umbellulone	1172	1167			Tr	9					
Undeca-1,3,5-triene	1173	1174						1.1			
Terpinen-4-ol	1175	1174	1.2	0.2	8.1	3.6			7.4	1.2	
m-Cymen-8-ol	1184	1176			Tr						
p-Cymen-8-ol	1186	1179	Tr		0.1	0.2			0.2	Tr	
α-Terpineol	1189	1186	0.1	0.1	0.4	1.1			0.6	0.1	
p-Menth-3-en-7-al	1191	1195		0.7							
cis-Piperitol	1194	1195			0.1	Tr					
Myrtenal	1190	1195							0.1		

Myrtenol	1194	1194			0.1		0.1			0.3		
Methyl chavicol	1199	1195										1.6
trans-Piperitol	1207	1207				0.1	Tr			0.2	Tr	
Verbenone	1208	1204					Tr			0.2		
di-sec-Butyl disulfide	1209	1212						0.8				
endo-Fenchyl acetate	1218	1218				0.2						
trans-Carveol	1219	1215					0.1			Tr		
Coahuilensol. methyl ether	1224	1219									0.2	
Citronellol	1233	1223				0.3	0.8				0.1	
Thymol. methyl ether	1233	1232		0.3		Tr	0.1		0.2			
Cumin aldehyde	1238	1238	2.9		30.2					Tr		
Carvacrol, methyl ether	1239	1241		21.9			Tr		0.1	Tr		
Carvone	1241	1239	38.4				Tr					
Disulfide, 2-butenyl 1-methylpropyl, (*E*)-	1249	1257						0.2				
Disulfide, methyl 1-(methylthio)propyl	1251	1265						0.1				
(4*Z*)-Decen-1-ol	1261	1255					0.1					
Piperitone	1253	1249				0.1	0.1				Tr	
Methyl citronellate	1265	1257									0.4	
trans-Ascaridol glycol	1265	1266								Tr		
trans-*p*-Menth-2-en-7-ol	1266	1268			0.2							
Isopulegyl acetate	1276	1275				Tr						
α-terpinen-7-al	1278	1283								Tr		
neoiso-3-Thujanol acetate	1279	1281									Tr	
(*E*)-Anethole	1284	1282	3.5		0.1							96.7
Bornyl acetate	1285	1287		0.1		0.2	0.4		0.1	0.3	36.3	
γ-Terpinen-7-al	1287	1290			17.3							
p-Cymen-7-ol	1289	1289			2.4							
trans-Sabinyl acetate	1294	1289							0.1		18.2	
Lavandulyl acetate	1295	1288						0.1				
Thymol	1298	1289		0.1	0.1							
trans-Pinocarvyl acetate	1298	1298						0.2				
Terpinen-4-ol acetate	1299	1299				0.5	0.3			0.1		
Carvacrol	1302	1298		Tr	Tr							
Methyl geranate	1327	1322									Tr	
p-Mentha-1,4-dien-7-ol	1328	1325			0.8							
trans-Carvyl acetate	1339	1339					Tr					

α-Terpinyl acetate	1344	1346					3.2	0.6		0.1	2.2		
α-Cubebene	1346	1345					0.3	Tr			0.1	Tr	
α-Copaene	1366	1374					0.1			0.3	0.1	Tr	
Daucene	1373	1380				0.1							
β-Cubebene	1381	1387								0.1	Tr	Tr	
β-Elemene	1383	1389					0.1			0.4	0.6		
Sibirene	1392	1400									0.3		
Longifolene	1394	1407						Tr				Tr	
(*E*)-Caryophyllene	1407	1417	14.7			Tr	Tr	0.2		0.5	0.2	Tr	
Methyl eugenol	1408	1403						Tr					
β-Copaene	1418	1430									Tr		
2,5-Dimethoxy-*p*-cymene	1421	1424								0.2			
cis-Thujopsene	1421	1429						0.1					
Z-Propenyl-*sec*-butyl trisulphide	1421	1433[f]							17.0				
E-Propenyl-*sec*-butyl trisulphide	1424	1435[f]							6.1				
γ-Elemene	1430	1434					Tr				0.4		
α-*trans*-Bergamotene	1431	1432				Tr							
cis-Muurola-3,5-diene	1440	1448					Tr	2.7			Tr	Tr	
α-Humulene	1440	1452	3.9					0.1	0.1	0.1	0.3	Tr	
trans-Muurola-3,5-diene	1443	1451										Tr	
cis-Cadina-1(6),4-diene	1456	1461					Tr	6.4					
(*E*)-β-Farnesene	1458	1454			Tr	0.1				Tr			
cis-Muurola-4(14),5-diene	1461	1465				Tr	1.2						
E and *Z*-Propenyl-1-(methylthio) propyl disulphide	1462	1465							0.9				
β-Acoradiene	1466	1469				0.1							
trans-Cadina-1(6),4-diene	1468	1475									Tr	0.1	
γ-Muurolene	1471	1478									Tr	0.1	
Germacrene D	1468	1484								0.6	0.9	0.2	
γ-Himachalene	1471	1481											0.6
β-Selinene	1474	1489									0.1		
ar-Curcumene	1481	1478					0.1	Tr			0.3		
trans-Muurola-4(14),5-diene	1483	1493									0.1	0.1	
Epishyobunone	1483	1497								1.7			
α-Selinene	1484	1498									0.1		
β-Alaskene	1489	1498						Tr					

Bicyclogermacrene	1490	1500					0.1				0.2		
α-Muurolene	1490	1500					Tr			0.1	0.1	0.2	
Epizonarene	1494	1501						1.6					
α-Zingiberene	1495	1493			Tr								0.1
Shyobunone	1504	1500								3.8			
α-Alaskene	1507	1512					0.1	0.1					
γ-Cadinene	1508	1513								0.2	0.8	0.3	
β-Curcumene	1510	1514					0.1						
Kessane	1513	1529								2.8			
δ-Cadinene	1513	1522					0.2	0.5		3	0.9	1.5	
trans-Calamenene	1516	1521						0.8					
Myristicin	1521	1517			0.5								
10-*epi*-Cubebol	1524	1533						Tr					
trans-Cadina-1,4-diene	1526	1533									Tr	Tr	
α-Cadinene	1532	1537										Tr	
α-Calacorene	1536	1542						0.1					
cis-Muurol-5-en-4-β-ol	1540	1550						0.1					
Elemol	1545	1548					0.1					1	
trans-Muurol-5-en-4-α-ol	1549	1559						0.2					
Germacrene B	1550	1559			Tr		Tr				0.5		
Elemicin	1563	1555			0.1								
(*E*)-Nerolidol	1663	1561					0.1						
Germacrene D-4-ol	1563	1574								0.2		0.1	
Spathulenol	1565	1577									0.6		
Caryophyllene oxide	1572	1583				Tr		0.1			0.1		
Salvial-4(14)-en-1-one	1581	1594									Tr		
Carotol	1586	1594				0.1							
Cedrol	1589	1600					Tr	0.1					
Humulene epoxide II	1599	1608						0.1			0.1		
β-Oplopenone	1601	1607										0.1	
1,10-*di*-*epi*-Cubenol	1607	1618						0.1					
1-*epi*-Cubenol	1621	1628									0.1	0.1	
α-Acorenol	1621	1632						0.5					
Dill apiole	1623	1620		3.3	17.5								
β-Acorenol	1626	1636						0.1					
epi-α-Cadinol	1629	1638					Tr	0.2		0.1	0.2	0.2	
epi-α-Muurolol	1635	1640					Tr				Tr	0.2	

β-Eudesmol	1641	1649										0.1	
α-Cadinol	1642	1652					Tr	0.3		Tr	0.1	0.3	
Shyobunol	1674	1688								0.3			
cis-14-*nor*-Muurol-5-en-4-one	1676	1688						0.3					
epi-α-bisabolol	1682	1683					0.2						
Germacra-4(15),5,10(14)-trien-1-α-ol	1694	1685					0.1						
(*Z*)-Nuciferol	1722	1724					Tr	0.1					
β-(*Z*)-Curcumen-12-ol	1755	1754					Tr						
(*E*)-pseudoisoeugenyl 2-methylbutyrate	1844	1841											0.4
Isohibaene	1905	1933					Tr						
Isophyllocladene	1944	1966					0.1						
Manool oxide	1974	1987					0.2						
13-*epi*-Manool oxide	1995	2009					Tr						
Nezukol	2091	2132					0.8	0.3					
Phyllocladanol	2172	2209					0.1						
Total identified (%)			99.8	97.1	99.3	99.6	98.7	99.4	93.2	97.1	99.3	99.6	99.4

[a] Linear retention index calculated on a HP-5MS column (30 m x 0.25 mm, 0.1 m f.t.) using a mixture of *n*-alkanes C_7-C_{30} (Supelco, Bellefonte, CA). [b] Retention index value taken from Adams (2007) or NIST 17. [c] Relative percentage values as a mean of three measurements (RSD% in all cases in the range 2-20%). [d] C.sa, *Cannabis sativa*; C.ca., *Carum carvi*; C.ma., *Crithmum maritimum*; C.cy., *Cuminum cyminum*; C.le; x *Cupressocyparys leylandii*; C.ar., *Cupressus arizonica*; F.a.f., *Ferula assa-foetida*; F.gu., *Ferula gummosa*; J.co., *Juniperus communis*; J.pf., *Juniperus x pfitzeriana*; P.an., *Pimpinella anisum*. [e] Tr, traces, % < 0.1. [f] Comparison with data of Kasaian et al. (2016).

References

Adams R (2007) Identification of Essential Oil Components by Gas Chromatography/ Mass Spectrometry, 4th ed. Allured Publishing Corp., Carol Stream, IL, USA.

Kasaian J, Asili J, Iranshahi M (2016) Sulphur-containing compounds in the essential oil of *Ferula alliacea* roots and their mass spectral fragmentation patterns. Pharm Biol 54:2264–2268.

NIST 17 (2017) Mass Spectral Library (NIST/EPA/NIH). National Institute of Standards and Technology, Gaithersburg, USA.
